# Epigenetic Modification as a Regulatory Mechanism for Spatiotemporal Dynamics of ANO1 Expression in Salivary Glands

**DOI:** 10.3390/ijms20246298

**Published:** 2019-12-13

**Authors:** Yonghwan Shin, Sang-Woo Lee, Eun Namkoong, Woojin An, Jong-Ho Lee, Peter D Brown, Kyungpyo Park

**Affiliations:** 1 Department of Physiology, School of Dentistry, Seoul National University and Dental Research Institute, Seoul 110-749, Korea; yonghwas@usc.edu (Y.S.); goodman23@snu.ac.kr (S.-W.L.); eunnamkoong@snu.ac.kr (E.N.); 2 Department of Biochemistry and Molecular Biology, Norris Comprehensive Cancer Center, University of Southern California, Los Angeles, CA 90033, USA; woojinan@usc.edu; 3 Department of Oral and Maxillofacial Surgery, Seoul National University Dental Hospital, Seoul 110-749, Korea; 4 Faculty of Biology, Medicine and Health, University of Manchester, Manchester M13 9PT, UK; Peter.D.Brown@manchester.ac.uk

**Keywords:** Anoctamin1, salivary gland, organogenesis, epigenetic regulation, DNA methylation

## Abstract

Anoctamin1 (ANO1), a calcium activated chloride channel, is known to play a critical role in salivary secretion. In the salivary gland, ANO1 is expressed exclusively in the acinar cells, with no expression in the ductal cells. However, the mechanisms that determine this distinctive cell type-dependent expression pattern of ANO1 remain unknown. In this study, we discovered that the cell-dependent expression of ANO1 during salivary gland organogenesis is regulated by DNA methylation of ANO1 CpG islands. ANO1 CpG islands in e12 embryonic submandibular glands (eSMG) are highly methylated, but those in e14 eSMG or adult SMG are significantly unmethylated. The differential expression pattern of ANO1 in duct and acini is defined at e14. Artificial demethylation by treatment with the demethylating agent 5-aza-2’-deoxycytidine (5-Aza-CdR), induced the expression of ANO1 in both the ductal cell line Human Submandibular Gland (HSG) and in the duct cells of adult mouse SMG. During the trans-differentiation in Matrigel of duct-origin HSG cells into acinar-like phenotype, significant demethylation of ANO1 CpG islands is observed. This may be due to the reduced expression of DNA methyltransferase (DNMT) 3a and 3b. These results suggest that the differential expression of ANO1 in salivary glands during organogenesis and differentiation is mainly regulated by epigenetic demethylation of the ANO1 gene.

## 1. Introduction

ANO1 is a Ca^2+^ activated Cl^−^ channel (CaCC), which is widely expressed in tissues such as sensory neurons, smooth muscle cells, and salivary glands. In salivary glands, ANO1 is one of the most important pathways for the apical Cl^−^ ion efflux required for fluid secretion [1]. Therefore, ANO1 is thought to be a potential target for the treatment of salivary gland dysfunction and/or the generation of fully functional artificial salivary gland tissue. In adult salivary glands, ANO1 is mainly expressed in the apical membrane of the secretory acinar cells, but not in the epithelial cells that line the ducts of the gland [2]. The mechanisms that determine this differential expression of ANO1 in the acinar and duct cells are still unknown. A previous study reported that ANO1 expression is regulated by epigenetic mechanisms involving DNA methylation of the ANO1 gene promoter region in head and neck squamous cell carcinomas, but similar studies have not been conducted on normal salivary gland tissue [3]. 

Epigenetic mechanisms, such as DNA methylation and histone modification, are known to be involved in the regulation of gene expression without requiring any changes in the DNA sequence [4,5]. The hypo- and hypermethylation pattern of CpG islands modulates global and site-specific gene expression by altering the degree of chromatin condensation. This subsequently determines the binding of transcription factors to the target genes [6]. The hypermethylation process is usually carried out by DNA methyltransferases (DNMTs) such as Dnmt1, Dnmt3a, and Dnmt3b [5,6,7,8]. Epigenetic patterning is crucial to the development and regeneration of various epithelial organs. For example, the differentiation of both liver and pancreas from the early endoderm is determined by epigenetic regulation of liver specific genes (*Alb1*, *Ttr*, and *Afp*), and an early pancreatic gene (*PDX1*), respectively [9]. Furthermore, during pancreatic islet development, the fates of alpha and beta cells are determined by epigenetic regulation, so that even after full differentiation both cell types can be trans-differentiated in response to epigenetic modulations [9]. The prostate gland is another example showing the importance of spatiotemporal methylation dynamics during organogenesis. Basal epithelium-specific DNA methylation of E-cadherin is required for proper budding and further branching morphogenesis of the prostate [10]. In salivary glands, however, there have been no studies of such developmental epigenetic dynamics. In this paper, we address this omission, by using isolated embryonic submandibular glands (eSMG) to investigate the potential epigenetic determinants of ANO1 expression during salivary gland organogenesis. 

Since tissue regeneration generally recapitulates the developmental process [11,12,13], revealing the mechanisms behind cell type-dependent expression of ANO1 during development may be helpful in in vitro efforts to generate salivary glands expressing functional ANO1. It is known that both salivary gland acinar and duct cells arise from primitive progenitor cell populations during salivary gland organogenesis, and that salivary gland ductal progenitor cells remaining in the adult intercalated ducts can differentiate into acinar cells during regeneration of damaged salivary glands [14]. Thus, it is possible that epigenetic modulation of gene expression is involved in the cell-type dependent expression of specific proteins associated with acinar and duct cells [15,16]. Our previous study revealed that epigenetic regulation is involved in the differential expression of Cystic fibrosis transmembrane conductance regulator (CFTR) in salivary gland acini and duct [17]. However, this work was limited to artificial epigenetic modulation in different kinds of immortalized cell lines by 5-aza-2’-deoxycytidine (5-Aza-CdR), a DNMT inhibitor consequently inducing DNA demethylation. In this study, the epigenetic regulation of ANO1 in salivary gland duct and acinar cells is examined under more physiologic conditions by using Matrigel-induced trans-differentiation of the Human Salivary Gland (HSG) cell line as model of development. The HSG cell line is derived from intercalated ducts of irradiated human submandibular glands, and is known to trans-differentiate into acinar cells when cultured on basement membrane extracts such as Matrigel or laminin-1 [18,19,20]. By using this and other experimental models, we demonstrate that the differential expression of ANO1 in acini and duct is regulated by epigenetic mechanisms. 

## 2. Results

### 2.1. Time-Dependent Changes of ANO1 Expression during Salivary Gland Organogenesis Are Epigenetically Determined

**Figure 1:**
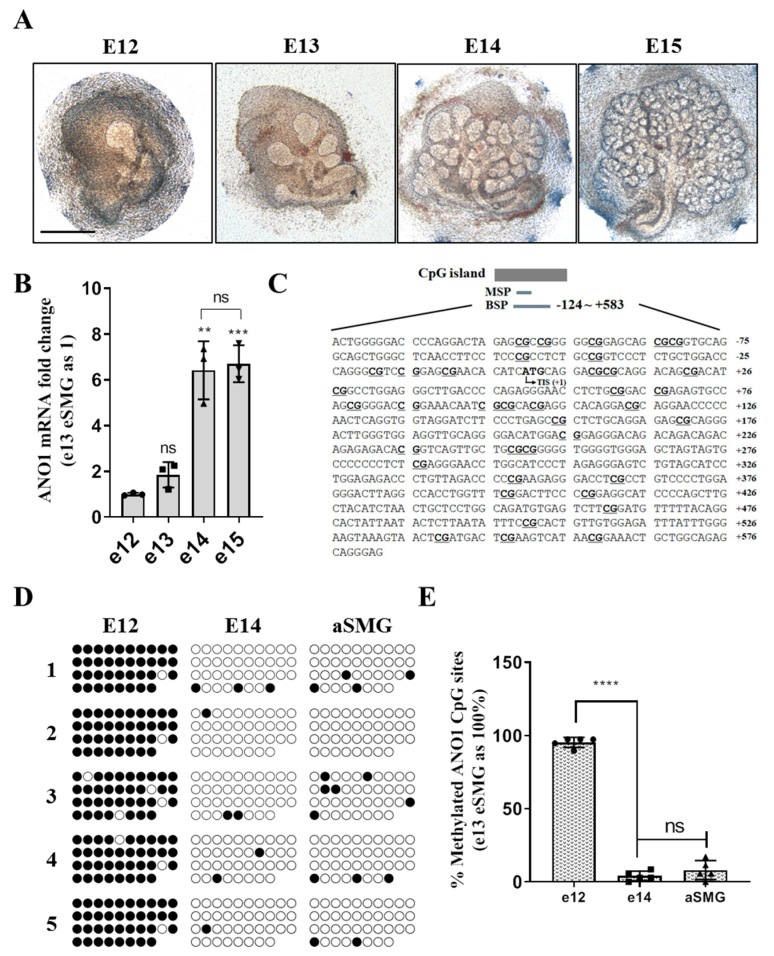
Time-dependent changes in ANO1 expression during salivary gland organogenesis are epigenetically regulated. (**A**) Bright field images of embryonic submandibular glands (eSMGs) isolated at each embryonic day 12 to 15 (E12 to E15). Each image is representative of four replicates. Scale bar = 500 µm. (**B**) Relative mRNA expression levels of ANO1 at each embryonic day (e12, 13, 14, and 15). mRNA was extracted from 20 eSMGs for each group. (**C**) The sequence of mouse ANO1 CpG island locus (National Center for Biotechnology Information (NCBI) accession: NC_000011, region: −124 to +573 (relative to start codon)). The ANO1 CpG islands are located at the positions containing 38CG (indicated by the underlined, bold text) and Transcription initiation site (TIS). (**D**) Bisulfite sequencing results of five separate clones for the ANO1 CpG islands from e12, e14, and adult SMG (aSMG). ○ = unmethylated cytosines, or ● = methylated cytosines. (**E**) The number of methylated CGs in e12, e14, and aSMGs is expressed as percentage of the total number of CGs (*n* = 5). Differences were determined by a one way ANOVA followed by Tukey’s multiple comparison test. **: *p* < 0.01; ***: *p* < 0.001; ****: *p* < 0.0001.

To explore the spatiotemporal pattern of ANO1 expression in developing salivary glands, embryonic submandibular glands (eSMGs) were harvested at e12 and e13. Some of the eSMGs were cultured ex vivo in order to obtain e14, and e15 eSMGs. Early embryonic salivary glands (e12) consist of only one epithelial bud, but they then radically expand to produce hundreds of buds with complex structures via a developmental process called “branching morphogenesis” (Figure 1A). The transcriptional expression level of ANO1 was minimal at e12 but significantly increased at e14 (Figure 1B). Our hypothesis was that epigenetic mechanisms help determine this change in ANO1 expression during salivary gland organogenesis. To test this hypothesis, bisulfite sequencing of CpG islands in ANO1 gene (Figure 1C) was performed. As shown in Figure 1D,E, CpG islands of ANO1 are highly methylated (95.7% methylation rate) at the early stage of organogenesis (e12). They are significantly demethylated (4% methylation), however, at the mid-late stage of organogenesis (e14), when they exhibit a similar degree of methylation to that seen in the adult salivary glands (7% methylation). These results indicate that epigenetic regulation may be a key mechanism in determining the time-dependent change of ANO1 expression during salivary gland organogenesis. 

### 2.2. Differential Expression of ANO1 in Acini and Duct of Embryonic and Adult Salivary Glands

**Figure 2:**
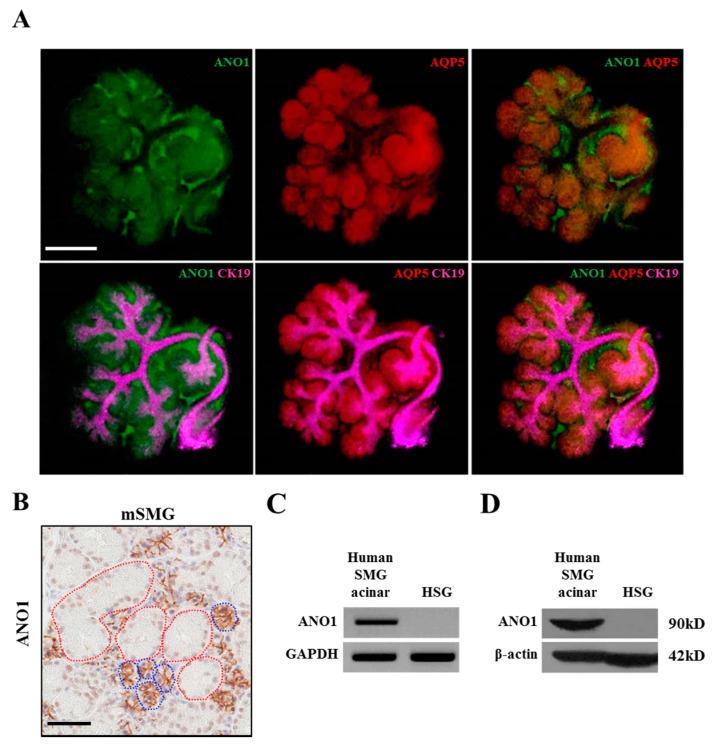
Differential expression of ANO1 in acinar and ductal cells of embryonic and adult salivary glands. (**A**) Immunostained images of e14 eSMGs were obtained by confocal microscope. ANO1 expression is shown in green. Acinar cells were identified by AQP5 expression (red), whereas ductal cells are characterized by CK19 expression (magenta). Merged images showing AQP5, ANO1, and CK19 are also displayed. Each image is representative of four replicates and the scale bar = 200 μm. (**B**) Immunohistochemistry of ANO1 (brown) in adult mouse SMGs (mSMG). Acinar cells (blue dotted lines), and duct cells (red dotted lines) are identified, with ANO1 expressed exclusively in the acinar cells. The image is representative of three replicates and the scale bar = 50 μm. (**C**) mRNA expression of ANO1 in human SMG acinar cells and HSG (ductal) cells by reverse transcription polymerase chain reaction (RT-PCR). The image is representative of 3 replicates. (**D**) Protein expression of ANO1 in human SMG acinar cells and Human Salivary Gland (HSG) cells by western blot. The image is representative image of 3 replicates.

To determine the expression of ANO1 in acini and duct cells during development, immunohistochemistry was performed on e14 eSMGs. Figure 2A shows ANO1 is mainly expressed in AQP5 positive (acinar) cells, but not in the K19 positive (ductal) cells. Figure 2B shows that this distinctive pattern of ANO1 expression is also observed in adult mouse SMGs, with ANO1 expressed only in acinar cell membranes and not in the duct cells (Figure 2B). Additionally, in human samples, ANO1 expression is detected in SMG acinar cells, but not in HSG cell line derived from human SMG ducts (Figure 2C,D). 

### 2.3. The Demethylation Agent (5-Aza-Cdr) Restores the Expression and Function of ANO1 in HSG Cells 

**Figure 3:**
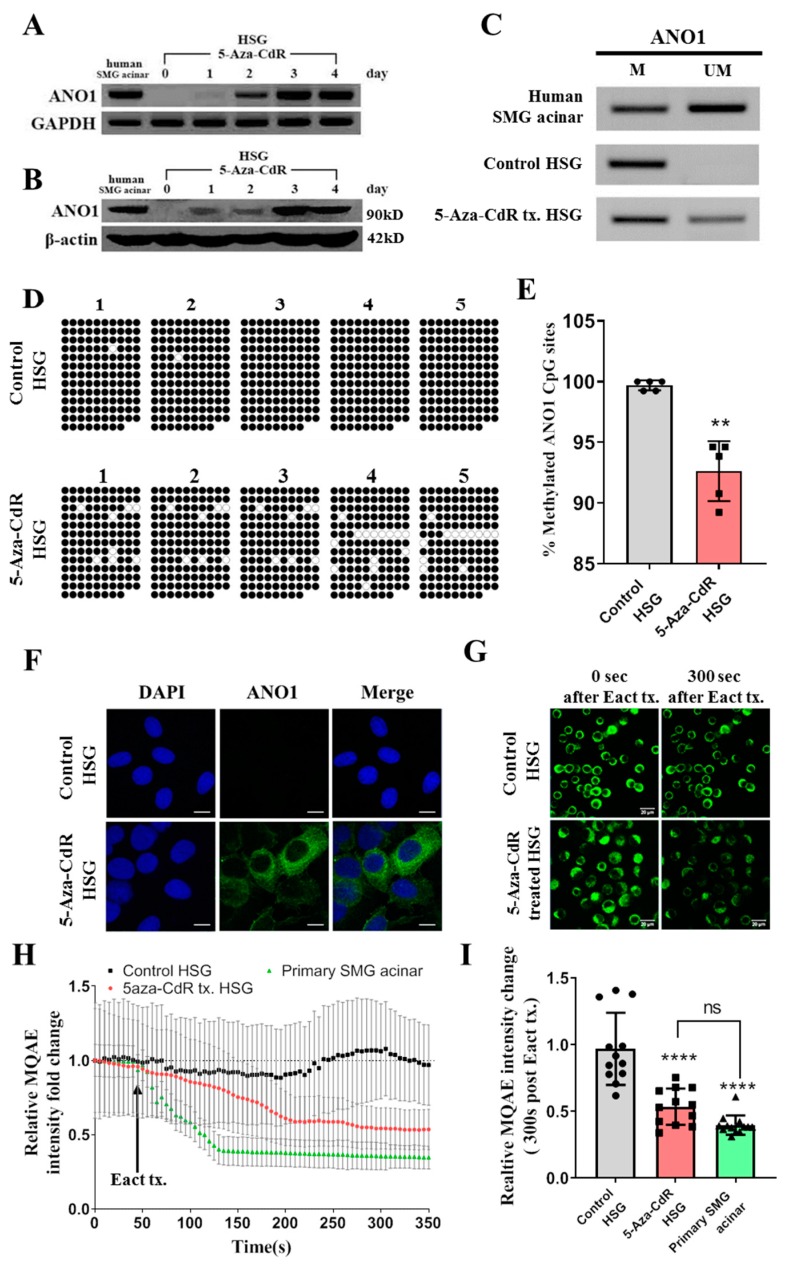
ANO1 expression and function in HSG cells treated with 5-Aza-CdR. (**A**) mRNA for ANO1 was determined in HSG cells treated with 10 μΜ 5-Aza-CdR for 1, 2, 3, and 4 days via RT-PCR. ANO1 mRNA is not detected before treatment (Day 0), but gradually increased after treatment with the 5-Aza-CdR (Days 1–4). The expression of mRNA for GAPDH was unaffected by 5-Aza-CdR. (**B**) ANO1 protein expression in HSG cells treated with 10 μΜ 5-Aza-CdR for 1, 2, 3, and 4 days. Protein expression increased with time after treatment with 5-Aza-CdR. No change in β-actin expression was observed. (**C**) Methylation-specific PCR (MSP) was performed with sodium bisulfite-modified genomic DNA obtained from human SMG acinar cells and HSG cells. M indicates PCR products of the methylated ANO1 CpG islands. UM indicates PCR products of the unmethylated ANO1 CpG islands. ANO1 CpG islands in control HSG cells were highly methylated, while a large proportion was unmethylated in human SMG acinar cells. ANO1 CpG islands in 5-Aza-CdR-treated HSG cells are partially unmethylated. Each image is a representative of three replicates. (**D**) Bisulfite sequencing results of five separate clones for the ANO1 CpG islands from Control HSG cells and 5-Aza-CdR-treated HSG cells. ○ = unmethylated cytosine, or ● = methylated cytosine. (**E**) Number of methylated CGs expressed as a percentage of the total number of CGs in Control HSG and 5-Aza-CdR-treated HSG cells. A decrease in methylation was observed in 5-Aza-CdR-treated HSG cells (*p* < 0.01). (**F**) Expression of ANO1 is visualized in control and 5-Aza-CdR-treated HSG cells by immunocytochemistry (Blue: DAPI, Green: ANO1). ANO1 expression was observed in both the cytosol and membrane after the 3-day treatment with 5-Aza-CdR. Each image is representative of three replicates and the scale bar = 15 μm. (**G**) Changes in intracellular Cl^-^ monitored by (6-Methoxyquinolinio)acetic acid ethyl ester bromide (MQAE) fluorescence in control and 5-Aza-CdR-treated HSG cells. Cells were stimulated with Eact an ANO1 agonist. Left panel: before Eact treatment, Right panel: 300 s after Eact treatment. Each image is representative of three replicates and the scale bar = 50 μm. (**H**) MQAE signals quantified from Figure 3G and Figure S3. Cells were stimulated with Eact after a 50 s control period. Data from untreated, control HSG cells are in black. Data from the 5-Aza-CdR-treated HSG cells are shown in red. Data from primary mouse SMG acinar cells are shown in green. (mean ± Standard Deviation (SD), *n* = 12). (**I**) Mean changes in MQAE fluorescence in control, 5-Aza-CdR-treated HSG cells, and primary mouse SMG acinar cells measured 300 s after the Eact stimulation. Intensities at 0 sec in each corresponding group were normalized to 1 (*n* = 12; *p* < 0.001). Statistical differences were determined by unpaired t-test for (**E**) or a one way ANOVA followed by Tukey’s multiple comparison test for (**I**). **: *p* < 0.01; ****: *p* < 0.0001.

To further test the hypothesis that the expression of ANO1 SMG cells is regulated epigenetically, the effects of a demethylation agent, 5-Aza-CdR, were determined on ANO1 expression in HSG cells. Figure 3A,B show that at Day 0, neither mRNA for ANO1 nor ANO1 protein was expressed in HSG cells. After treatment with 10 μM 5-Aza-CdR for 1, 2, 3, and 4 days, however, expression of mRNA and ANO1 protein gradually increased (Figure 3A,B, Figure S2A,B). On the third day of 5-Aza-CdR treatment ANO1 expression in HSG cells becomes equivalent to that in human SMG acinar cells (Figure 3A,B). Therefore, in all subsequent experiments, a 3-day treatment with 5-Aza-CdR was employed. 

The role of demethylation in the recovery of ANO1 expression in 5-Aza-CdR-treated HSG cells was next examined in experiments using: (i) methylation-specific polymerase chain reaction (MSP) and (ii) bisulfite sequencing of bisulfite converted-128 CGs in human ANO1 CpG islands (Figure S1). Results of the MSP analysis indicates that both methylated and unmethylated ANO1 CpG islands are observed in human SMG acinar cells. By contrast, only methylated ANO1 CpG islands were observed in the HSG cell line (Figure 3C). Treatment of HSG cells with 5-Aza-CdR, resulted in ANO1 CpG islands which were partially demethylated (Figure 3C). Bisulfite sequencing was performed to quantify methylation of ANO1 CpG islands in untreated and treated HSG cells. Figure 3D,E show that 99.7% of ANO1 CpG islands are methylated in control HSG cells, but the methylation level is significantly reduced to 92.6% in 5-Aza-CdR-treated HSG cells (*p* < 0.01).

Immunostaining was performed to determine whether this level of demethylation is sufficient to permit the recovery of ANO1 expression. ANO1 was shown to be robustly expressed in both the membranes and cytosol of the 5-Aza-CdR-treated HSG cells (Figure 3F). By contrast, no ANO1 expression was observed in untreated HSG cells (Figure 3F). To test whether the membrane-localized ANO1 is functional the movement of chloride ions (Cl^−^) using (6-Methoxyquinolinio) acetic acid ethyl ester bromide (MQAE). MQAE is an intracellular fluorescence Cl^−^ sensor for which fluorescence is quenched by Cl^−^, thus, the signal intensity is reduced by an increase in intracellular Cl^−^ activity [21]. To stimulate Cl^−^ movement via ANO1 cells were stimulated by N-aroylaminothiazole “activators” (Eact), a specific agonist for ANO1 [22]. In both primary mouse SMG acinar cells and 5-Aza-CdR-treated HSG cells, Eact caused a marked decrease in MQAE fluorescence within 150 sec, indicating an increase in the intracellular Cl^−^ concentration (Figure 3G,H, Figure S3). However, no change in intracellular Cl^−^ concentration was observed in control HSG cells. Approximately 300 s after Eact treatment, MQAE signal intensity reached a plateau, and at this point, the MQAE signal intensity of each group was compared quantitatively. The signal intensity was significantly decreased in 5-Aza-CdR-treated HSG cells compared to control HSG cells (Figure 3I). It is of note that although primary mouse SMG acinar cells showed faster ion influx than 5-Aza-CdR-treated HSG cells, the plateau values after 300 s were similar (Figure 3I). These results suggest that the functional expression of ANO1 is regulated by DNA methylation of ANO1 CpG islands.

### 2.4. Expression of ANO1 is Regulated by Methylation in Adult Mouse SMG 

**Figure 4:**
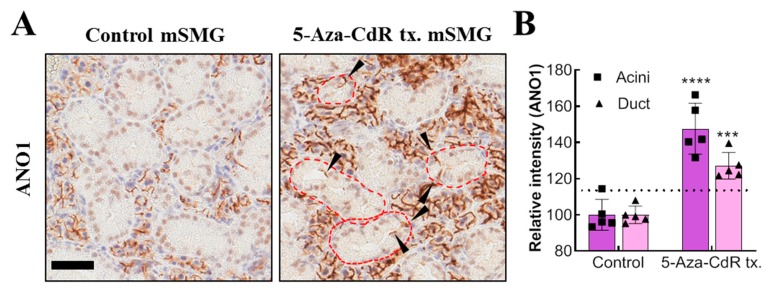
Expression of ANO1 is regulated by methylation in adult mouse SMG. (**A**) ANO1 expression (brown) in SMG of mice either treated with PBS or a high dose 5-Aza-CdR (25 mg/kg was determined by immunohistochemistry (5 mice for each group). Duct structures in 5-Aza-CdR tx. mouse SMG are marked by the red-dotted line. ANO1 expression observed in the duct of treated mice is indicated by the black arrows. Scale bar = 50 μm. (**B**) Intensity of ANO1 expression in acini and duct of the 5-Aza-CdR treated mice from the immunohistochemistry is quantified relative to control (*n* = 5). ANO1 expression is partially restored in duct after the seven day-consecutive 5-Aza-CdR-treatment. Control as 100%. Unpaired t-test was performed. ***: *p* < 0.001, ****: *p* < 0.0001.

The role of epigenetic modulation of ANO1 expression in acini and duct cells was next examined in vivo. Mice were injected intraperitoneally with a PBS, low (2.5 mg/kg) or high dose (25 mg/kg) of 5-Aza-CdR for 7 consecutive days. Immunohistochemical data, show that in control mouse SMG (mSMG), there is a distinctive expression pattern of ANO1, with no ANO1 expression in the ducts, as previously described in Figure 2B (Figure 4A, left panel). However, ANO1 was expressed in some duct cells harvested from high dose 5-Aza-CdR treated mice observed (Figure 4A, right panel, black arrow). Furthermore, ANO1 expression in acinar cells was also significantly elevated in the mice treated with the high dose. Thus, ANO1 expression of both salivary gland acinar and ductal cells appears to be regulated by gene methylation (Figure 4A,B). No changes in ductal or acinar ANO1 expression were observed in low dose 5-Aza-CdR-treatment group (Figure S4).

### 2.5. Trans-Differentiation of Duct-Origin Cells into Acinar Cells Restores ANO1 Expression via Epigenetic Mechanisms.

**Figure 5:**
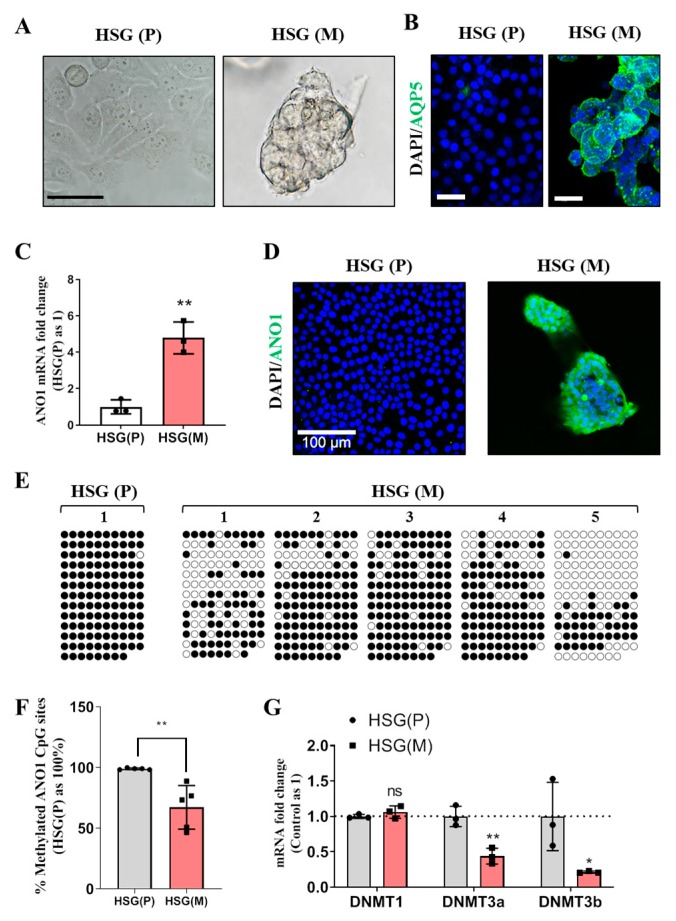
Trans-differentiated HSG cells grown in Matrigel exhibit ANO1 expression restored by epigenetic mechanisms. (**A**) Bright field images of HSG cells cultured on plastic culture dish (HSG (P)) and Matrigel (HSG (M)). The HSG (M) grows as an acinar-like structure. Each image is representative of three replicates and the scale bar = 20 μm. (**B**) Immunofluorescence of AQP5 expression in HSG (P) and HSG (M). AQP5 is detected only in HSG (M). Each image is representative of three replicates, and the scale bar = 20 μm. (**C**) mRNA for ANO1 measured by quantitative polymerase chain reaction (qPCR), is significantly higher in HSG (M) compared to HSG (P) (*n* = 3). (**D**) ANO1 protein expression visualized by immunocytochemistry is observed in HSG (M), but not in HSG (P). Each image is representative of three replicates, and the scale bar = 100 μm. (**E**) HSG (M) shows decreased methylation by bisulfite sequencing. Data are from five separate clones of ANO1 CpG islands from HSG (m) and one representative HSG (P) clone (data from a total of 5 HSG (P) clones are presented in Figure S3). ○ = unmethylated cytosines, or ● = methylated cytosines. (**F**) Decrease in the number of methylated CGs in HSG (M) compared to HSG (P) (p < 0.01). Data are expressed as a percentage of the total number of CGs (*n* = 5). (**G**) mRNA expression of DNMT 1, 3a, and 3b. Expression in HSG (M) is normalized with respect to expression in HSG (P) (*n* = 3 for each). Significant decreases in the expression of DNMT3a and DNMT2b were observed in HSG (M). For DNMT1, expression was unchanged. All differences were determined by t-tests for unpaired data, where: ** *p* < 0.01 and * *p* < 0.05.

When grown encapsulated in Matrigel, HSG cells (HSG (M)), trans-differentiate to produce an acinar cell-like morphology [19] (Figure 5A). This change in morphology in HSG (M) cells is accompanied by the expression of aquaporin 5, an acinar marker, after three days in Matrigel (Figure 5B). Whereas there is negligible aquaporin 5 expression in HSG cells cultured on plastic surface (HSG(P)) for three days (Figure 5B). At the same time, expression of mRNA for ANO1 is significantly elevated in HSG(M) groups (Figure 5C), while immunostaining shows the strong ANO1 protein expression in HSG(M) cells (Figure 5D). Bisulfite sequencing results revealed that ANO1 CpG islands in HSG(M) cells are partially, but significantly demethylated (average methylation = 67.2%) compared to 98.9% in HSG(P) cells (Figure 5E,F, Figure S5). To further scrutinize the potential mechanism of demethylation, changes in the expression of three DNA methylatransferase (DNMT) enzymes (DNMT1, DNMT3a, and DNMT3b) were monitored. The expression of mRNA for DNMT1 was unchanged in HSG(M) cells. However, mRNA expression for both DNMT3a and DNMT3b were significantly decreased in HSG(M) groups compared to HSG(P) groups (Figure 5F). In summary, CpG islands of ANO1 in HSG cells are originally hypermethylated, but become hypomethylated when the cells trans-differentiate into the acinar-like phenotype, possibly through the down regulation of the enzymes DNMT3a and DNMT3b.

## 3. Discussion

In this study, various experimental models including: eSMG, mouse SMG, and in vitro trans-differentiation HSG cell line have been used to obtain evidence for epigenetic regulation of ANO1 expression in the development of SMGs. ANO1 CpG islands in e12 eSMG are highly methylated, but those in e14 eSMG or adult SMG are significantly unmethylated. The differential expression pattern of ANO1 in duct and acini is defined at e14. When artificial demethylation is induced by treatment of 5-Aza-CdR, both duct-origin HSG cell line and adult mouse SMG duct cells exhibited the expression of ANO1. During the trans-differentiation of duct-origin HSG cells into acinar cells within Matrigel, significant demethylation of ANO1 CpG islands is observed, confirming that the expression of ANO1 in salivary glands is mainly regulated by epigenetic modulation. 

DNA methylation is involved in various cellular processes including embryonic development, regulation of gene expression, and determination of chromosomal structure and activity [8]. During early developmental stages, the degree of DNA methylation is also highly elevated to map the specific regulatory patterns of gene promoter regions required for further developmental processes [8]. Therefore, precise DNA methylation patterning is essential for maintaining normal organogenesis. Although there has been no study on global epigenetic patterning during organogenesis of salivary glands, unpublished data from this laboratory show that the treatment of 5-Aza-CdR to ex vivo cultured e13 eSMG completely abolished branching morphogenesis and induced rapid degeneration of epithelium (data not shown). This is because demethylation by 5-Aza-CdR is non-specific, thus, we are only able to observe epigenetic regulation of ANO1 during organogenesis, rather than modulating it. In addition, since the current study is limited to the negative modulation of ANO1′s methylation status by using 5-Aza-CdR, it will be necessary to confirm our hypothesis with more advanced epigenetic modulation system such as dCas9-DNA methyltransferase MQ1 fusion protein which targets specific gene to produce methylation [23]. Furthermore, the molecular cues which initiate methylation or demethylation of ANO1 CpG islands during salivary gland organogenesis or trans-differentiation of HSG cells also remain to be elucidated. 

Based on our results from trans-differentiating HSG cells into acinar cells, it can be speculated that downregulation of DNMTs by basement matrix may be responsible for demethylation of ANO1 during organogenesis. This hypothesis can be supported by a classic methodology, the epithelial rudiment culture technique, which has been routinely used for studying salivary gland branching morphogenesis. Epithelial rudiments of eSMG without mesenchyme can branch and differentiate within Matrigel [24]. However, since Matrigel is heterogeneous ECM mixtures isolated from Engelbreth-Holm-Swarm mouse sarcoma [25], key ECM substances triggering the demethylation of ANO1 CpG islands in HSG cells should be further specified. One study reports that the expansion of c-kit+ epithelial progenitor cells during salivary gland organogenesis is epigenetically regulated by a microRNA secreted from mesenchyme [26]. Therefore, it is also possible that the demethylation of ANO1 CpG islands can be triggered by single or multiple mesenchymal factors including miRNAs, soluble growth factors, and extracellular matrix substances.

In salivary glands, the Cl^−^ channels CFTR and ANO1 are known to play a major role in salivary secretion [2,27]. CFTR is a cyclic AMP-activated Cl^−^ channel, whereas ANO1 is a Ca2+-activated Cl^−^ channel [2,27,28,29]. CFTR is predominantly expressed in SMG ducts but not in acinar cells, and in a previous study, we demonstrated that this differential expression is due to the DNA methylation of CFTR CpG islands [17]. By contrast, ANO1 is expressed in SMG acinar cells but not in the ducts [2,27]. Although differential expression patterns of ANO1 or CFTR in acini and duct are explained by epigenetic modification, not all acini or duct-specific proteins are regulated by epigenetic mechanisms. In the case of AQP5, a representative marker for the salivary gland acinar cell, their mRNA transcripts exist in HSG cells cultured on plastic surface, but because of an unknown mechanism, the AQP5 mRNA cannot be translated into functional proteins [20]. However, when they are cultured in Matrigel, the AQP5 mRNAs are translated into functional proteins [20]. Therefore, although AQP5 is exclusively expressed in acini like ANO1, it is not regulated by epigenetic mechanisms. 

From a tissue-engineering perspective, restoring expression of acini-specific functional proteins in ductal cells by using the clinically available demethylating agent, 5-Aza-CdR, has enormous therapeutic potential for salivary gland dysfunction. Radiation-induced xerostomia is one of the most common complications caused by head and neck cancer radiotherapy [30,31]. Although the mechanisms are not yet fully understood, radiation significantly destroys acini while not harming ducts [32]. Therefore, one study suggests delivering the Aquaporin-1 gene to duct cells using a viral vector for the restoration of salivary flow [33,34]. Likewise, if it is possible to induce expression of acini-specific functional proteins in the remaining duct cells by using 5-Aza-CdR; they may replace the functions of acinar cells. In this study, continuous injection of 5-Aza-CdR successfully induces ANO1 in duct cells, showing the potential application of demethylating agents for the treatment of salivary gland dysfunction. It is notable that treatment of 5-Aza-CdR also increases ANO1 expression in acinar cells, which may lead to enhancement of salivation, but this requires further examination. 

The mechanism for the significantly different degree of expressional increase in duct and acinar ANO1 by in vivo injections of high dose 5-Aza-CdR also still needs to be explained. This might be due to the difference in physiological methylation levels in duct and acinar cells and insufficient 5-Aza-CdR delivery to submandibular glands. In fact, mice treated with low dose 5-Aza-CdR showed no such increased expression of ANO1 in acini or duct, suggesting that there is a threshold concentration for 5-Aza-cdR to achieve sufficient demethylation for the significant expressional increment of ANO1. Since high dose 5-Aza-CdR has potential risk of leukopenia or bone marrow hypoplasia [35], targeted delivery of concentrated 5-Aza-CdR via advanced drug delivery system may more efficiently recover ductal ANO1 expression with minimal systemic toxicity. There are several studies used continuous 5-Aza-CdR injections in vivo to correct non-cancerous diseases. For examples, altered methylation status of matrix metalloproteinase-9 and tissue inhibitor of metalloproteinase 1, 2 in injured kidney was corrected by series in vivo injection of 5-Aza-CdR, resulting in amelioration of renal fibrosis in mice [36]. Another possible application is cell therapy using in vitro-cultured immortalized salivary gland cell lines. Although HSG cell line is derived from neoplastic cells, it is a well-established candidate for the generation of artificial salivary glands [37]. First, HSG cells can differentiate into acinar cells when cultured in Matrigel [20,38]. Second, they functionally express muscarinic and purinergic receptors to increase intracellular calcium like primary salivary gland cells [39,40]. If expression of other acini-specific proteins can be induced by treatment of 5-Aza-CdR in HSG cultured without Matrigel, less immunogenic and more economic materials will be able to replace Matrigel. Therefore, global expressional changes of such genes should be examined via Next Generation Sequencing or proteome array after an artificial epigenetic modulation. It is also noted that the activity of ANO1 restored by 5-Aza-CdR in HSG cells seems lower than that of primary mouse SMG acinar cells, indicating that other channels or proteins expressed on primary acinar cells may have synergistic effect on ANO1 activity.

In summary, this study indicates that epigenetic modification by DNA methylation is a key regulatory mechanism for silencing and upregulation, respectively, of ANO1 expression in salivary gland acini and duct. Considering the importance of ANO1 in salivary secretion, an epigenetic approach to enhance or restore ANO1 expression has therapeutic potentials for the treatment of salivary gland dysfunction. 

## 4. Material and Methods

### 4.1. Cell Culture

Human submandibular gland cells, HSG, were grown in Dulbecco’s modified Eagle’s medium (DMEM) supplemented with 10% fetal bovine serum and 1% penicillin and streptomycin. Media was changed every 48 h, and the cells were passaged every 72 h. The cells were cultured at 37 °C in a humidified atmosphere of 95% air and 5% CO2. 

### 4.2. Matrigel Culture of HSG 

Growth factor-reduced Matrigel (Corning, Corning city, NY, USA) was thawed on ice. 10,000 HSG cells were mixed with 500 μL of the Matrigel on ice, and then, 20 μL Matrigel-HSG mixture were pipetted on to cell culture plates. The plates were incubated in 37 °C in a humidified atmosphere of 95% air and 5% CO2 for 15 min to solidify the Matrigel/HSG mixture. DMEM supplemented with 10% fetal bovine serum and 1% penicillin and streptomycin was slowly added on the Matrigel dome. The cells were cultured at 37 °C in a humidified atmosphere of 95% air and 5% CO2.

### 4.3. 5-Aza-CdR Treatment and Reverse Transcription-Polymerase Chain Reaction (RT-PCR)

HSG cells were treated with 10 μM 5-Aza-CdR for 1, 2, 3, and 4 days, and the medium containing 5-Aza-CdR was changed daily. Total RNA was isolated from untreated and 5-Aza-CdR treated HSG cells using the Trizol reagent according to the manufacturer’s instructions (Ambion, Life Technologies, Carlsbad, CA, USA). The purity and concentration of the total RNA were measured using a NanoDrop 1000 (Thermo Fisher Scientific, Waltham, MA, USA). Total RNA was also isolated by the same method from: human submandibular gland acinar (human SMG acinar) cells (see below), A253, and SGT cells. 

cDNA was synthesized from 1 μg of total RNA with reverse transcriptase. Primer sequences for ANO1 amplification and GAPDH used as an endogenous control were as follows: ANO1: forward 5′-AACCACACCCTCTCCTCCTT-3′ and reverse 5′-CTTTGGTGTTGTGGTGGTTG-3′; GAPDH: forward 5′-GAAGGTGAAGGTCGGAGTC-3′ and reverse 5′-GAAGATGGTGATGGGATTTC-3′. cDNA was applied as a template for ANO1 amplification, and the RT-PCR conditions were as follows: 95 °C for 5 min; followed by 35 cycles of denaturation at 94 °C for 30 s, annealing at 56 °C for 30 s, and extension at 72 °C for 30 s; and a final step at 72 °C for 10 min. The PCR products were separated by 1% agarose gel electrophoresis.

### 4.4. Western Blot Analysis

Cells were washed with phosphate buffered saline (PBS), harvested, and dissolved in a lysis buffer. The protein samples were separated using 8% sodium dodecyl sulfate polyacrylamide gel electrophoresis (SDS-PAGE) and transferred to a nitrocellulose membrane. After blocking with 10% non-fat milk (Seoul Milk, Seoul, Korea), the membrane was incubated at 4 °C with a primary rabbit antibody ANO1 (Santa Cruz Biotechnology, Santa Cruz, CA, USA, 1:1000), and then washed with Tris buffered saline + Tween 20 (TBST). The membrane was then incubated with an HRP-conjugated secondary antibody (Santa Cruz Biotechnology, Dallas, TX, USA, 1:5000) for 1 h at room temperature, which was visualized using an Enhanced chemiluminescence (ECL) reagent (Thermo Fisher Scientific, Waltham, MA, USA).

### 4.5. Intracellular Cl^−^ Ion Flux Monitoring 

Intracellular Cl^−^ ion was monitored by modified version of the method of Choi et al. [21]. Briefly, HSG cells were harvested from culture dish and loaded with 10mM MQAE dissolved in DMEM for 30 min at 37 °C. The cells were washed three times with HBSS, and attached to a cover-glass coated with Cell-Tak (Corning, NY, USA). The cover-glass formed the bottom of the experimental chamber. Fluorescence was monitored using a LSM700 Confocal Laser Scanning Microscope, fitted with a filter cube No. 49 (Ex/Em = 365/445, Zeiss, Oberkochen, Germany). Baseline intensity calibration was measured for 50 s. After that, cells were treated with 10 μM Eact, and fluorescence changes measured for a further 300 s at 5 s intervals. 

### 4.6. Ex vivo SMG Organ Harvesting and Immunostaining

Embryonic SMGs (eSMGs) from Institute of Cancer Research (ICR) mice were harvested and stained according to previously described methods [41]. Fetal mice were collected from e12 and 13 embryonic sacs and the eSMGs isolated. The isolated eSMGs were placed on Nucleopore filters (0.1 µm, Little Chalfont, Buckinghamshire, UK) floating on phenol red-free DMEM/F12 (1:1) containing 1% Pen/Strep, 0.15 µg/µL transferrin, and 5 ng/µL L-ascrobic acid. e12 and e13 eSMGs were stabilized for two hours or further cultured to obtain e14 and 15 eSMGs. After the incubation, bright field images were obtained by Nikon Ti digital inverted fluorescence microscope (Shinagawa, Tokyo, Japan). eSMGs at e14 stage were fixed with 4% paraformaldehyde and washed with PBS tween 0.5%. The fixed eSMGs were permeabilized with 0.1% Triton X-100 and blocked in PBS Tween 0.5% containing 10% donkey serum and 3% bovine serum albumin (BSA). The eSMGs were incubated overnight with primary antibodies against: ANO1 (1:200; Santa Cruz Biotechnology), Aquaporin 5 (1:200; Alomone Labs, Israel), or Cytokeratin 19 (1:200; DSHB, Iowa city, IA, USA). After a further overnight incubation with appropriate secondary antibodies conjugated with either: Cy2, Cy3, or Cy5, the eSMGs were mounted on slides. Immunocytochemistry staining was analyzed using an LSM700 Laser Confocal Scanning Microscope (Carl Zeiss, Oberkochen, Germany). The 3 × 3 image stitching mode was used to obtain images of whole eSMGs.

### 4.7. Immunofluorescence Staining of HSG Cells

HSG cells were grown on cell culture slides and treated with 10 μM 5-Aza-CdR (Sigma Aldrich, St. Louis, MO, USA) for 3 days. After treatment with 5-Aza-CdR, the HSG cells were fixed in 4% paraformaldehyde for 10 min and washed with PBS. For Matrigel-encapsulated HSG cells (3-day culture), they were fixed in ice cold 4% paraformaldehyde for 30 min and washed with PBS. Cells were permeabilized with 0.1% Triton X-100 for 10 min. Cells were then blocked with 10% donkey serum and 3% BSA for 60 min and washed with cold PBS/Glycine. After that, cells were incubated overnight with an anti-human ANO1 (1:200, Abcam, ab53212, Cambridge, UK) or anti-human AQP5 primary (1:200, Alomone Labs, #AQP-005, Hadassah Ein Kerem, Jerusalem, Israel) antibody at 4°C. After washing with PBS, the cells were incubated with an Alexa Fluor^®®^ 488 donkey anti-mouse or anti-rabbit IgG secondary antibody (1:200) and DAPI (1:1000) for 2 h at room temperature. HSG cells treated with or without 5-Aza-CdR were mounted with FLUO-GEL WITH TRIS BUFFER (Electron Microscopy Science, Hatfield, PA, USA) and visualized by an LSM700 (Carl Zeiss, Oberkochen, Germany). 

### 4.8. Immunohistochemistry of Adult Mouse SMG

Adult mouse SMGs in paraffin block sections were de-paraffinized and hydrated. For the de-paraffinization step, the sections were incubated in dry oven at 60C for 1 h and de-waxed in xylene five times for 4 min. The mouse SMG sections were rehydrated in 100%, 95%, and 75% ethanol for 3 min each and immersed in tap water for 5 min. For the antigen retrieval step, the sections were immersed into citrate buffer (0.01 M, pH 6.0) and microwaved at high, medium, and low power for 5 min each. After that, the sections were immersed in cold PBS. Next, to quench endogenous peroxidase, the sections were immersed in peroxidase-blocking solution (Agilent DAKO, S2023, Santa Clara, CA, USA) and washed three times with PBS for 5 min each. The sections were then incubated with anti-ANO1 primary antibody (100:1, Abcam, ab532132) for 30 min. The sections were washed in PBS three times, and incubated with EnVision +systems-HRP labelled polymer anti-rabbit (Agilent DAKO, K4003) for 30 min. The sections were washed in PBS three times for 5 min each. After that, the sections were incubated in DAB solution (Agilent DAKO, GV825) for 5 min, and the reaction was stopped by washing in tap water. For the counterstaining, the sections were immersed in Mayer’s hematoxylin solution for 1 min. After the dehydration step (immersing in 75%, 80%, 95%, and 100% ethanol for 1 min each), the slide sections were cleared in xylene 4 times for 5 min each. Finally, the slide sections were mounted with Permount Mounting Medium (Fisher chemical, SP15-100, Waltham, MA, USA) for further analysis. 

### 4.9. Quantification of ANO1 Expression from Immunohistochemistry of Adult Mouse SMG

Immunohistochemistry images of adult mouse SMG stained with anti-ANO1 antibody (Abcam, ab532132) were digitally scanned by Aperio AT2 (Leica, Wetzlar, Germany). The scanned images were then captured by Aperio ImageScope version 12.3.2.8013 (Leica, Wetzlar, Germany). The ANO1 expression in duct and acini was quantified by SABIA ver 1.0.0.0 (Solution for Automatic Bio-Image Analysis, Seoul, Korea). In detail, areas of duct or acini were manually selected based on their morphological difference, and then, the relative ANO1-positive areas were automatically calculated within the previously selected area. 

### 4.10. Animal Experiments 

Eight-week old ICR mice were purchased from Daehan BioLink (Choong-buk, Korea). Mice were injected intraperitoneally with either PBS or low (2.5 mg/kg)/high dose (25 mg/kg) 5-Aza-CdR for 7 consecutive days. The mice were then sacrificed and the SMGs are harvested. The animal experiment protocol used in this study was approved by Seoul National University Institutional Animal Care and Use Committee (Approval number: SNU-190610-5-1, Approval date: 17 June 2019).

### 4.11. Methylation-Specific PCR

Genomic DNA was isolated using the QIAamp DNA Blood Mini Kit (Qiagen, Venlo, Netherlands) from: HSG cells (before and after treatment with 5-Aza-CdR), human SMG cells, A253 cells, and SGT cells. For methylation-specific PCR (MSP), bisulfite modification of genomic DNA was performed with the EpiTect Bisulfite Kit (Qiagen) as described previously [17]. MSP was performed with bisulfite-treated genomic DNA using specific primers for the methylated or unmethylated forms of the ANO1 CpG islands. The following methylation-specific primer sequences were used: M forward 5′-TTTTAAGGTAAAGGCGGGTC-3′ and M reverse 5′-CTCGATACGAAAAACGCCTA-3′; U forward 5′-TATTTTTAAGGTAAAGGTGGGTT-3′ and U reverse 5′-CTCAATACAAAAAACACCTAAAC-3′.

### 4.12. Bisulfite Sequencing

2 μg of genomic DNA was modified by a sodium bisulfite conversion reaction with the EpiTect Bisulfite Kit (Qiagen) according to the manufacturer’s instructions. The modified genomic DNA was amplified with bisulfite primer sequences of the ANO1 CpG island locus (NCBI accession: NC_000011, region: 70078169 to 70079120, 952 bp) designed by the Methyl Primer Express^TM^ software v1.0 as follows: forward 5′-AAAAATAAAATTTGGAGGGGTT-3′ and reverse 5′-CCCTAACTACCCCAACAAATAC-3′. The PCR reactions were carried out as follows: 94 °C for 5 min; 35 cycles of 94 °C for 45 s and 55 °C for 45 s; and a final cycle of 72 °C for 45 s at the end. The PCR products were purified with a Gel Extraction Kit (Qiagen) and ligated into the pGEM-T easy vector (Promega, Madison, WI, USA). Five separate clones each from HSG cells, eSMGs, and adult SMGs were selected for bisulfite sequencing analysis according to the previously described method [17].

### 4.13. Human and Mouse Submandibular Gland (SMG) Acianr Cell Isolation

Human SMG tissues were obtained from the patients visiting the Seoul National University Dental Hospital (SNUDH) from 2016 to 2018. Biopsied tissues were confirmed as normal SMG tissue by pathology department of SNUDH. Mouse SMGs were harvested from 8-week old male ICR mice. SMG tissues were immersed in dissociation buffer (RPMI supplemented with 1 mg/mL Collagenase IV and 1mg/mL DNase 1) and placed in the Gentle MACS machine (Miltenyi Biotech, North Rhine-Westphalia, Germany). By using a preprogrammed protocol (M_impTumor3) in Gentle MACS, the SMG tissues were mechanically dissociated and incubated in 37 °C water bath for 30 min. This step was repeated once more, and dissociated SMG cells were centrifuged at 1500 rpm for 5 min. After washing with HBSS three times, dissociated SMG cells were filtered through 70 μm strainer (Miltenyi Biotech, North Rhine-Westphalia, Germany). Acinar cells were isolated from the dissociated SMG cells by density gradient cell separation method using Accudenz described by Xin Xu et al. (Accurate Chemical and Scientific Corporation, Westbury, NY, USA) [42]. All ethical guidelines and consent forms were approved by Institutional Review Board of SNUDH (Approval number: CRI11023G, approval date: 5 July 2011). 

### 4.14. RT-PCR

mRNAs in Human SMG acinar cells, mouse eSMG, and HSG cells were extracted by Trizol reagent (Invitrogen, Carlsbad, CA, USA). cDNA was prepared from 2μg of total RNA using reverse transcriptase with oligo-dT primer. The RT-PCR was performed with specific primer for ANO1. The primer sequences are as follows: For human ANO1, forward 5′-AACCACACCCTCTCCTCCTT-3′ and reverse 5′-CTTTGGTGTTGTGGTGGTTG-3′. For mouse ANO1, forward 5′-GAGGCCAGTAGCCATCAGAG-3′ and reverse 5′-GAGAGCGTGTGATTGACGAA-3′. For GAPDH, forward 5’-GAAGGTGAAGGTCGGAGTC-3’ and reverse 5’-GAAGATGGTGATGGGATTTC-3′. The RT-PCR was performed with a specific condition: 35 cycles of denaturation at 94 °C for 40 sec, annealing at 55 °C for 40 sec, and extension at 72 °C for 40 sec, with a final extension at 72 °C for 10 min. cDNA from samples were amplified using PCR amplification machine (PTC-1148C, Bio-Rad Laboratories Inc., Hercules, CA, USA). The RT-PCR products were loaded in 1.2% agarose gels (Sigma Aldrich, St. Louis, MO, USA) supplemented with 0.1 μg/mL ethidium bromide (Sigma Aldrich, St. Louis, MO, USA). Gel-running was performed by an electrophoresis machine (Mupid -2 plus, OPTIMA, Tokyo, Japan). Finally, PCR bands in the gel were visualized by UV light of bioimaging system (TS-312R, Spectoline, Westbury, NY, USA). 

### 4.15. Statistical Analysis

All experiments were repeated in triplicate. Statistical analysis was performed by unpaired t-test or one-way ANOVA with Tukey’s multiple comparisons test, using Graph Pad Prism 8 software (GraphPad Software, Inc., La Jolla, CA, USA); p-values less than 0.05 were considered as statistically significant.

## Supplementary Materials

Supplementary materials can be found at https://www.mdpi.com/1422-0067/20/24/6298/s1.
