# Supplementary material for: Epigenetic Modification as a Regulatory Mechanism for Spatiotemporal Dynamics of ANO1 Expression in Salivary Glands
Source: Int J Mol Sci. 2019 Dec 13;20(24):6298. doi: 10.3390/ijms20246298 (PMC6940850; doi:10.3390/ijms20246298)
Supplement: Supplementary file 1 [file ijms-20-06298-s001.pdf]

Supplementary Materials

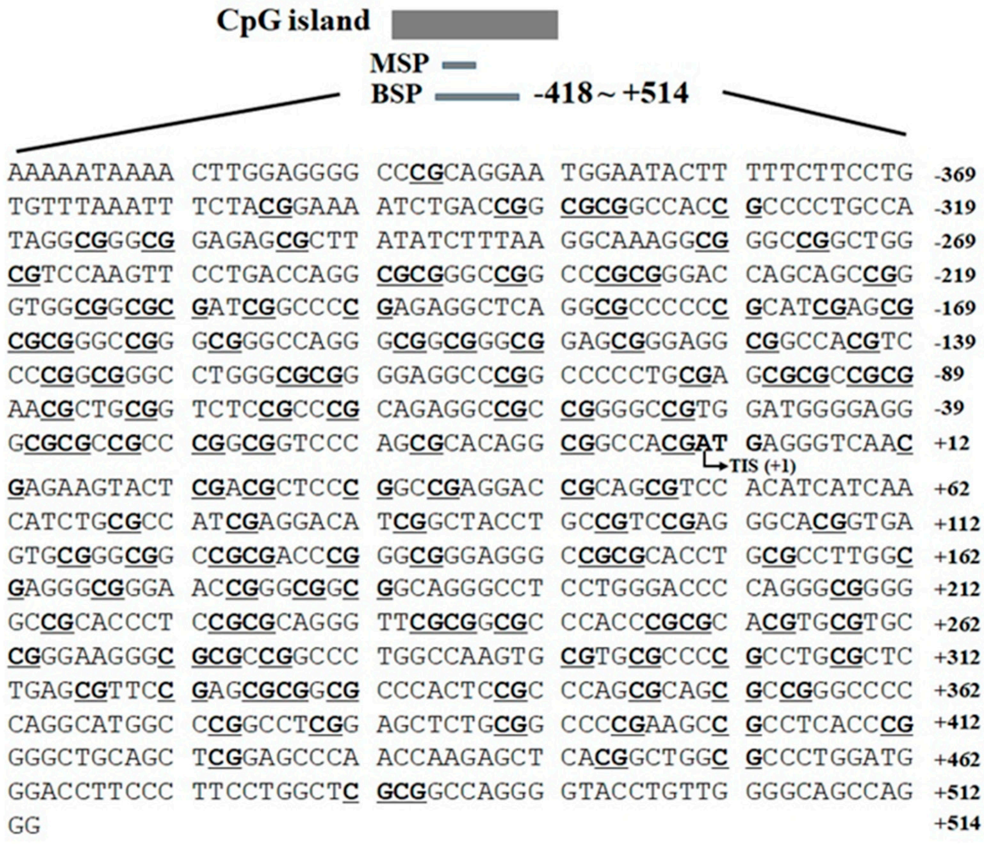

Figure S1. The sequence of the ANO1 CpG island locus (NCBI accession: NC_000011, region: 70078169 to 70079120). The ANO1 CpG islands were located at the positions containing 128 CG (underlined and bold) and TIS (bold).

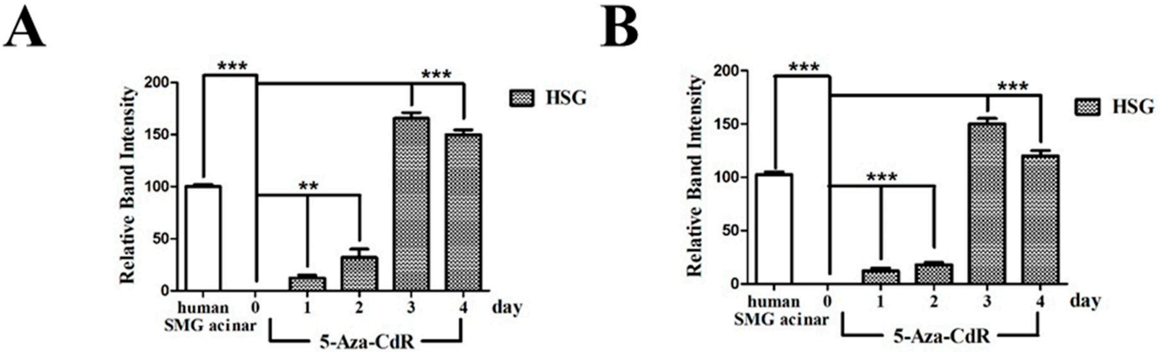

Figure S2. (**A**) Intensities of qPCR bands from Figure 3A quantified by ImageJ. ANO1 expression significantly increases in HSG cells after one-day of treatment with 5-Aza-CdR. A Three-day treatment increases ANO1 expression to a level comparable with that of human SMG acinar cells. Data are expressed relative to the human SMG cells (n = 3). Statistical analysis was by one way ANOVA, followed by Tukey's multiple comparisons test. *: $p < 0.05$, **: $p < 0.01$, ***: $p < 0.001$. (**B**) Intensities of western blot bands from Figure 3B quantified by ImageJ. Treatment with 5-Aza-CdR significantly increases ANO1 expression in HSG cells. Data are expressed relative to the human SMG cells (n = 3). *: $p < 0.05$, **: $p < 0.01$, ***: $p < 0.001$.

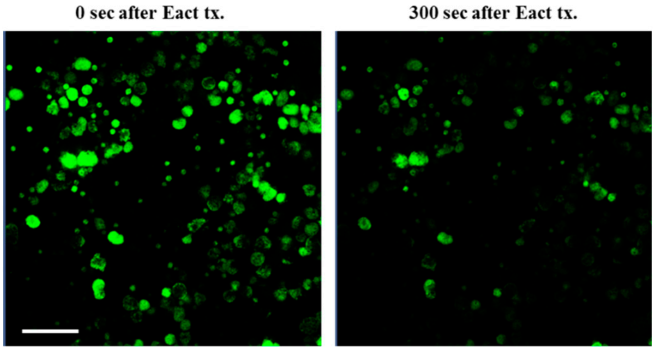

Figure S3. Primary acinar cells isolated from mouse SMG were subjected to MQAE-based Cl^- ion influx monitoring triggered by Eact treatment. Scale bar = 50 μm

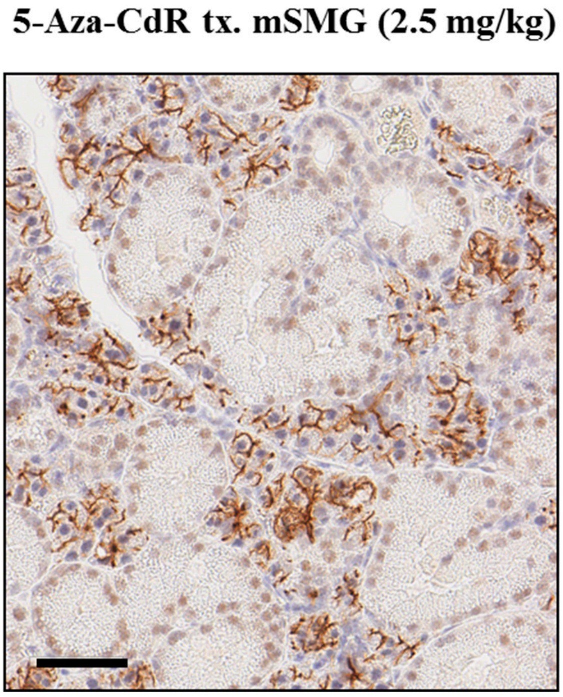

Figure S4. ANO1 expressions on SMG of mice treated with low dose 5-Aza-CdR (2.5 mg/kg) are visualized as dark brown color by immunohistochemistry (3 mice for each group). Scale bar = 50 μm.

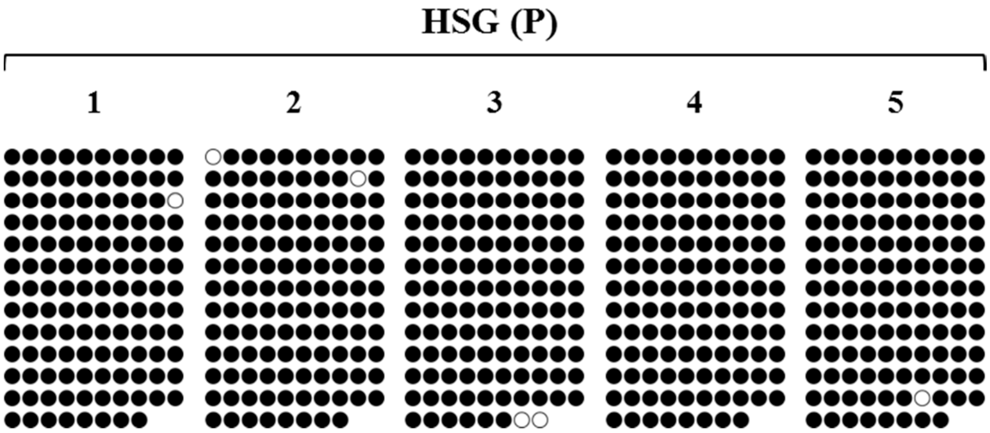

Figure S5. Bisulfite results of five clones of HSG cells cultured on plastic surface (HSG (P)). ○ = unmethylated cytosines, or ● = methylated cytosines.
